# Consistent and Efficient Modeling of the Nonlinear Properties of Ferroelectric Materials in Ceramic Capacitors for Frugal Electronic Implants

**DOI:** 10.3390/s20154206

**Published:** 2020-07-28

**Authors:** Yves Olsommer, Frank R. Ihmig

**Affiliations:** Fraunhofer Institute for Biomedical Engineering, Department of Biomedical Microsystems, 66280 Sulzbach, Saar, Germany; frank.ihmig@ibmt.fraunhofer.de

**Keywords:** ferroelectric materials, hysteresis, Mathcad, ANSYS, electronic implants, inductive coupling, computing time, memory consumption

## Abstract

In recent years, the development of implantable electronics has been driven by the motivation to expand their field of application. The main intention is to implement advanced functionalities while increasing the degree of miniaturization and maintaining reliability. The intrinsic nonlinear properties of the electronic components, to be used anyway, could be utilized to resolve this issue. To master the implementation of functionalities in implantable electronics using the nonlinear properties of its electronic components, simulation models are of utmost importance. In this paper, we present a simulation model that is optimized in terms of consistency, computing time and memory consumption. Three circuit topologies of nonlinear capacitors, including hysteresis losses, are investigated. An inductively coupled measurement setup was realized to validate the calculations. The best results were obtained using the Trapezoid method in ANSYS with a constant step size and a resolution of 500 k points and using the Adams method in Mathcad with a resolution of 50 k points. An inductive coupling factor between 7% and 10% leads to a significant improvement in consistency compared to lower coupling factors. Finally, our results indicate that the nonlinear properties of the voltage rectifier capacitor can be neglected since these do not significantly affect the simulation results.

## 1. Introduction

During the last decade, implantable electronics have become increasingly popular for the treatment of drug-resistant diseases and as an alternative to traditional therapies using pharmaceuticals. A large number of implantable electronics, such as the retinal implants Argus II (Second Sight Medical Products Inc., Sylmar, CA, USA), IRIS II (Pixium Vision S.A., Paris, France), Alpha AMS and IMS (Retina Implant AG, Reutlingen, Germany) [1,2], the vagus nerve stimulators AspireSR and SenTivaTM (LivaNova PLC, London, UK) [3], or the hypoglossal nerve stimulator from Inspire Medical Systems [4], are nowadays used to treat diseases such as retinitis pigmentosa, age-related macular degeneration, epilepsy, depression, pain, tinnitus and obstructive sleep apnea.

The extension of the field of application of implantable electronics is associated with increased requirements in terms of functionality and miniaturization without impairing reliability. Most of the implantable electronic devices with application in functional electrostimulation comprise a large number of active electronic components, sensors and a bulky battery unit. As a consequence, the degree of miniaturization is restricted, whereby the implantable electronics cannot be placed at the location where the stimulation pulses need to be applied. The electrical stimulation pulses are delivered through wire-bound electrodes, which are susceptible to migration and fracture over time [5]. Examples include the Argus II epiretinal implantable system [1,6] and the Alpha IMS subretinal implantable system [1,7] where a wire connection through the eye is required to connect the stimulation electrodes to the implantable electronics. Such connections are exposed to mechanical stress, require a more complex surgical procedure and increase the risk of infections. The latter can lead to complications and prevent long-term use [8]. From this point of view, highly miniaturized implantable systems would be more suitable [9].

A considerable advantage of advanced implantable electronics is the implementation of a wide range of functionalities. This is at the expense of high circuit complexity and the need of a battery unit, which may lead to malfunctions and age-related battery replacement [10]. One solution concept is to use only passive electronic components to increase the degree of miniaturization and to use the intrinsic nonlinear properties of these electronic components to realize certain functionalities.

By applying this principle of frugal engineering, the stimulation current in implantable electronics could be determined by using the nonlinear junction capacitance of a rectifier diode without having to use sensors or other active electronic components [11]. This principle was also used in the so-called “Neural Dust” sensors to wirelessly acquire neural signals using the intrinsic properties of a piezo-element [12]. Due to the considerable reduction in the overall number of electrical components, a high degree of miniaturization was achieved. As a result, an implantable sensor with a length of 3 mm and a cross-section of 1 mm^2^ was produced. The implantable electronics considered in this paper contain neither batteries nor sensors or active electronic components and are therefore not suitable for autonomous operation. Power is supplied by induction at a frequency below 1 MHz using an extracorporeal wearable device. The amount of inductively transferred power directly impacts the induced voltage.

In recent years, the suitability of ferroelectric ceramic capacitors, as control elements of resonant half-bridge converters and as tuning elements of resonant circuits for wireless power transmission, has been investigated [13,14,15,16,17,18]. In these applications, the intrinsic nonlinear properties of ferroelectric ceramic capacitors were used in resonant circuits. The use of ferroelectric ceramic capacitors as a control element and tuning element was realized by setting a DC bias voltage. In contrast, our strategy is to drive the nonlinear capacitors with an AC voltage.

In a previously published conference paper, we have introduced a simulation model for modeling the nonlinear properties of ferroelectric materials in ceramic capacitors [19]. Exemplary calculations of two serially connected nonlinear capacitors were carried out in Mathcad Prime 3.1 and ANSYS 2019 R2 Simplorer and were subsequently validated by measurements. As a result, it was found that the Adams, Bulirsch–Stoer, Backward Differentiation Formula, Radau5 and the fourth-order Runge–Kutta method with adaptive step size and a resolution of 50 k points (Mathcad) and the Adaptive Trapezoid-Euler method with constant step size and a resolution of 500 k points and with an adaptive step size and a resolution between 50 k and 5 M points (ANSYS) are most suitable. Using these calculation methods, the modeling in ANSYS and Mathcad showed small and equal deviation from the measurements [19].

Despite the high agreement between the calculations and the measurements, discrepancies in amplitude and time constants have been observed. In this paper, the cause of these discrepancies is investigated with the aim of improving the simulation model in terms of consistency, computing time and memory consumption.

## 2. Methods

**Figure 1:**
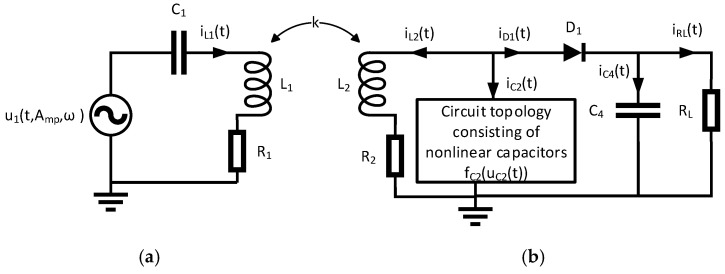
Representation of the inductively coupled system for power transmission: (**a**) Primary side consisting of an ideal voltage source $u_{1}\left(t,A_{mp},\omega \right)$ and a series resonant circuit consisting of a capacitor C_1_, an inductance L_1_ and a loss resistor R_1_; (**b**) Secondary side consisting of a parallel resonant circuit, which is composed of the inductance L_2_, a circuit topology consisting of nonlinear capacitors f_C2_(u_C2_(t)) and the loss resistance R_2_, a rectifier consisting of the diode D_1_ and the capacitor C_4_ and an ohmic load R_L_ resulting from the biological tissue and electrode properties. The inductive coupling between the primary and secondary inductances is represented by the coupling factor k.

The designed circuit consists of an “extracorporeal” primary side that represents the inductive power supply (Figure 1a) and an “implantable” secondary side that converts the inductively received power into stimulation pulses (Figure 1b). The concept of the circuit in Figure 1b is based on the design of the first visual prosthetic implant from Brindley [20,21,22,23]. Both resonant circuits are tuned to the same frequency. Power is transmitted on this frequency for a defined pulse duration and at a defined interval between successive pulses. The stimulation pulses are generated by rectification of the individual power pulses with diode D_1_ and capacitor C_4_. The duration and interval between the individual power pulses corresponds to the stimulation duration and frequency at the electrode impedance R_L_.

The inductively coupled system for power transfer represented in Figure 1 was described by the first-order differential Equations (1)–(9) [24]:

(1) $$L_{1}\cdot \frac{d}{dt}i_{L1}\left(t\right)+R_{1}\cdot i_{L1}\left(t\right)+k\cdot \sqrt{L_{1}\cdot L_{2}}\cdot \frac{d}{dt}i_{L2}\left(t\right)+u_{C1}\left(t\right)=u_{1}\left(t,A_{mp},\omega \right),$$

(2) $$i_{L1}\left(t\right)=C_{1}\cdot \frac{d}{dt}u_{C1}\left(t\right),$$

(3) $$L_{2}\cdot \frac{d}{dt}i_{L2}\left(t\right)+R_{2}\cdot i_{L2}\left(t\right)+k\cdot \sqrt{L_{1}\cdot L_{2}}\cdot \frac{d}{dt}i_{L1}\left(t\right)=u_{C2}\left(t\right),$$

(4) $$i_{C2}\left(t\right)=f_{C2}\left(u_{C2}\left(t\right)\right)\cdot \frac{d}{dt}u_{C2}\left(t\right),$$

(5) $$i_{C4}\left(t\right)=C_{4}\left(u_{C4}\left(t\right)\right)\cdot \frac{d}{dt}u_{C4}\left(t\right),$$

(6) $$u_{C2}\left(t\right)=u_{D1}\left(t\right)+u_{C4}\left(t\right),$$

(7) $$i_{L2}\left(t\right)+i_{C2}\left(t\right)+i_{D1}\left(u_{D1}\left(t\right)\right)=0,$$

(8) $$i_{D1}\left(u_{D1}\left(t\right)\right)=i_{C4}\left(t\right)+i_{RL}\left(t\right),$$

(9) $$i_{RL}\left(t\right)=\frac{u_{C4}\left(t\right)}{R_{L}},$$

where:

k: inductive coupling factor between the inductances *L*_1_ and *L*_2_

$A_{mp}$: amplitude of the sinusoidal voltage $u_{1}\left(t,A_{mp},\omega \right)$

ω: angular frequency of the sinusoidal voltage $u_{1}\left(t,A_{mp},\omega \right)$

$i_{L1}\left(t\right)$: electrical current across the primary resonant circuit

$u_{C1}\left(t\right)$: electrical voltage across the capacitor *C*_1_

$i_{L2}\left(t\right)$: electrical current across inductance *L*_2_ and its loss resistance *R*_2_

$i_{C2}\left(t\right)$: electrical current across the circuit topology consisting of nonlinear capacitors $f_{C2}\left(u_{C2}\left(t\right)\right)$

$u_{C2}\left(t\right)$: electrical voltage across the circuit topology consisting of nonlinear capacitors $f_{C2}\left(u_{C2}\left(t\right)\right)$

$u_{D1}\left(t\right)$: electrical voltage across diode *D*_1_

$i_{D1}\left(u_{D1}\left(t\right)\right)$: electrical current flowing through the diode *D*_1_ as a function of the voltage $u_{D1}\left(t\right)$

$u_{C4}\left(t\right)$: electrical voltage across the capacitor *C*_4_

$i_{C4}\left(t\right)$: electrical current across the capacitor *C*_4_

$i_{RL}\left(t\right)$: electrical current across the resistive load *R_L_*

**Figure 2:**
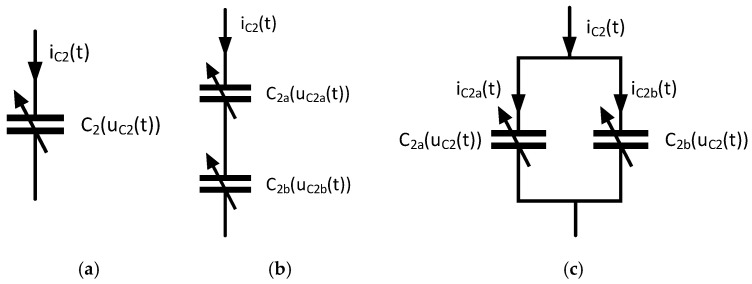
Circuit topologies with nonlinear capacitors: (**a**) one nonlinear capacitor, C_2_; (**b**) two serially connected nonlinear capacitors, C_2a_ and C_2b_; (**c**) two nonlinear capacitors, C_2a_ and C_2b_, connected in parallel.

We investigated the following structures of nonlinear capacitors shown in Figure 2. Depending on the structure under investigation, Equations (1)–(9) must be adapted.

Using the structure shown in Figure 2a, Equation (4) must be changed to Equation (10).

(10) $$i_{C2}\left(t\right)=C_{2}\left(u_{C2}\left(t\right)\right)\cdot \frac{d}{dt}u_{C2}\left(t\right),$$

By using the structure shown in Figure 2b, Equation (4) must be changed to Equations (11) and (12) and the voltage $u_{C2}\left(t\right)$ must be replaced by $u_{C2a}\left(t\right)+u_{C2b}\left(t\right)$.

(11) $$i_{C2}\left(t\right)=C_{2a}\left(u_{C2a}\left(t\right)\right)\cdot \frac{d}{dt}u_{C2a}\left(t\right),$$

(12) $$i_{C2}\left(t\right)=C_{2b}\left(u_{C2b}\left(t\right)\right)\cdot \frac{d}{dt}u_{C2b}\left(t\right),$$

By application of the structure shown in Figure 2c, Equation (4) must be changed to Equations (13)–(14) and the current $i_{C2}\left(t\right)$ must be replaced by $i_{C2a}\left(t\right)+i_{C2b}\left(t\right)$

(13) $$i_{C2a}\left(t\right)=C_{2a}\left(u_{C2}\left(t\right)\right)\cdot \frac{d}{dt}u_{C2}\left(t\right),$$

(14) $$i_{C2b}\left(t\right)=C_{2b}\left(u_{C2}\left(t\right)\right)\cdot \frac{d}{dt}u_{C2}\left(t\right),$$

### 2.1. Characterization of the Voltage Dependency of Ceramic Capacitors

**Figure 3:**
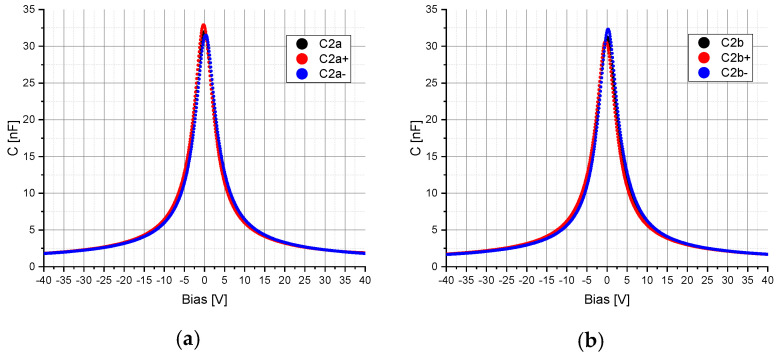
Measured electrical capacitance C of the capacitor: (**a**) C_2a_; (**b**) C_2b_. The electrical capacitance of C_2a_ and C_2b_ for a change in the bias voltage from −40 V to +40 V is represented by C_2a+_ and C_2b+_ and for a change in the bias voltage from +40 V to −40 V by C_2a−_ and C_2b−_. C_2a_ and C_2b_ corresponds to the averaged capacitances of C_2a+_ and C_2a−_ and C_2b+_ and C_2b−_ over the entire bias voltage range.

**Figure 4:**
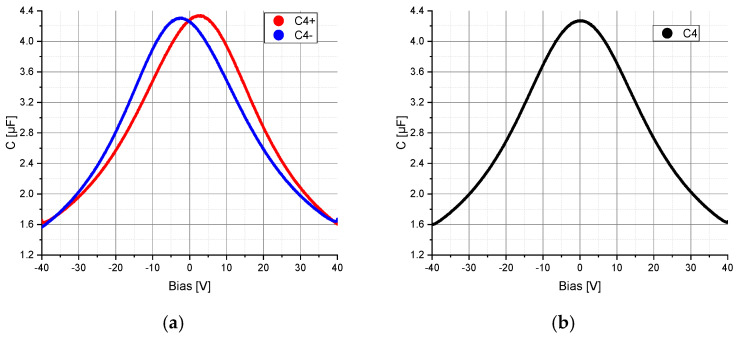
Measured electrical capacitance C of the voltage rectifier capacitor C_4_: (**a**) C_4+_ corresponds to the electrical capacitance of C_4_ for a change in the bias voltage from −40 V to +40 V and C_4−_ corresponds to the electrical capacitance of C_4_ for a change in the bias voltage from +40 V to −40 V; (**b**) Averaged capacitance of C_4+_ and C_4−_ over the entire bias voltage range.

The voltage dependency of the capacitors C_2_, C_2a_, C_2b_ and C_4_ was measured using the precision impedance analyzer Agilent 4294A (Agilent Technologies, Inc., Santa Clara, PA, USA, 4294A R1.11 Mar 25 2013) and the test fixture Agilent 16034E (Agilent Technologies, Inc., Santa Clara, PA, USA). The AC component was set to a frequency of 375 kHz for the capacitor C_2_, C_2a_ and C_2b_ and to 40 Hz (lower limit of the impedance analyzer) for the voltage rectifier capacitor C_4_. The amplitude was set to 5 mV and was superimposed with a DC bias voltage varying in the range from −40 V to +40 V with a resolution of 801 points. To determine the hysteresis, the electrical capacitance of C_2_, C_2a_, C_2b_ and C_4_ was measured by varying the bias voltage from −40 V to +40 V and from +40 V to −40 V. The obtained characteristic curves of the capacitors C_2_, C_2a_, C_2b_ and C_4_ were implemented in the simulation model in Mathcad (PTC, Boston, MA, USA) and ANSYS (ANSYS, Inc., Canonsburg, PA, USA) in order to include the voltage-dependent capacitance change in the calculations (Figure 3 and Figure 4). Additional specifications for the capacitors C_2_, C_2a_, C_2b_ and C_4_ can be found in Section 2.4.

### 2.2. Calculations in Mathcad Prime 3.1

We used the first-order differential Equations (1)–(14) (Section 2) in Mathcad Prime 3.1 to model the circuit shown in Figure 1 with the circuit topologies of nonlinear capacitors shown in Figure 2. To solve these differential equations, the Adams, Bulirsch–Stoer, and Runge–Kutta methods of fourth-order for non-stiff systems, and the Backward Differentiation Formula and Radau5 method for stiff systems, were applied. The tolerance of the calculations was set to 10^−7^ and the number of points for a given solution interval was set to 50 k, 500 k and 5 M. The step size was constant or varying within a solution interval, depending on the solver used. Under consideration of the currents i_C2_(t) or i_C2a_(t) and i_C2b_(t), the hysteresis losses can be incorporated into the model. The characteristic curves of the capacitors C_2_, C_2a_, C_2b_ and C_4_ have been interpolated with third order B-spline functions. In order to achieve different modulations of the electrical capacitance resulting from each circuit topology, the amplitude of the sinusoidal excitation $u_{1}\left(t,A_{mp},\omega \right)$ was varied from 0.1 V to 10 V in 0.1 V steps at a coupling factor k of 1% and 10%

### 2.3. Calculations in ANSYS 2019 R3 Simplorer

The “extracorporeal” primary side and the “implantable” secondary side were modeled in ANSYS 2019 R3 Simplorer according to Figure 1. The solvers based on the Euler, Adaptive Trapezoid-Euler and Trapezoid method were used. The number of points for a given solution interval was set to 50 k, 500 k and 5 M, with a constant and adaptive step size. For an adaptive step size, the number of points for the given solution interval is determined by the solver and can vary between 50 k and 5 M. In order to achieve different modulations of the electrical capacitance resulting from each circuit topology, the amplitude of the sinusoidal excitation $u_{1}\left(t,A_{mp},\omega \right)$ was varied from 0.1 V to 10 V in 0.1 V steps at a coupling factor k of 1% and 10%.

### 2.4. Model Validation by Means of a Measurement Setup

The simulation results were validated using a measurement setup. The components L_1_ and R_1_, C_1_ as well as L_2_ and R_2_ and C_4_ were measured with the precision impedance analyzer Agilent 4294A and the test fixture HP 1604D (Hewlett Packard, Palo Alto, CA, USA). For all circuit topologies in Figure 2, L_1_ and R_1_ (14.53 µH, 0.4 Ω, Würth Elektronik), and C_1_ (12.45 nF, WIMA, FKP1, 2 kV) remain constant.

A linear capacitor C_2_ (47 nF, 200 V, C0G), a nonlinear capacitor C_2_ (47 nF, ±20%, 4 V, X5R, 01005) and an inductance L_2_ and loss resistance R_2_ (3.76 µH, 0.3 Ω, Würth Elektronik) were used for the circuit topology shown in Figure 2a. The capacitors C_2a_ and C_2b_ (47 nF, ±20%, 4 V, X5R, 01005) and the inductance L_2_ and loss resistance R_2_ (8.45 µH, 0.86 Ω, Würth Elektronik) were used for the circuit topology shown in Figure 2b. Finally, the capacitors C_2a_ and C_2b_ (47 nF, ±20%, 4 V, X5R, 01005) and the inductance L_2_ and loss resistance R_2_ (1.75 µH, 0.28 Ω, Würth Elektronik) were used for the circuit topology in Figure 2c.

The electrical properties of the components D_1_ (MULTICOMP, 1N4148WS.) and R_L_ (1 kΩ, ±1%) were taken from the datasheets. The capacitors C_2_ (47 nF, 200 V, C0G), C_2a_ and C_2b_ (47 nF, ±20%, 4 V, X5R, 01005) and C_4_ (4.7 µF, 50 V) were determined according to Section 2.1 (Figure 3 and Figure 4).

Different voltages across the capacitors C_2_, C_2a_, C_2b_ and C_4_ were set by changing the distance between the inductances L_1_ and L_2_ on the primary and secondary sides. A loose coupling between the inductances L_1_ and L_2_ was ensured, so that the detuning of the resonant circuits on the primary and secondary sides was avoided in order to be able to compare the calculations and the measurements. The voltage Uc2RMS, resulting from the root mean square value over time of the voltage u_c2_(t) across the circuit topology consisting of nonlinear capacitors, and the voltage Uc4Mean, resulting from the mean value over time from the voltage u_c4_(t) at the load R_L_, were measured with the digital oscilloscope RIGOL MSO4054 (RIGOL Technologies, Inc., Suzhou, China). It should be noted that the measurement was performed on the internal memory and not on the graphical memory, otherwise the root mean square value would be wrong, due to insufficient resolution. The internal memory was accessed using the UltraSigma and UltraScope programs (RIGOL Technologies, Inc., Suzhou, China). The measured values refer to a time span of 14 ms, with a sampling rate of 4 GS/s. Furthermore, a pulsed inductive power transfer at a frequency of 375 kHz, a duration of 5 ms and a period of at least 1 s was performed, so that the thermal detuning of the capacitors C_2_, C_2a_, C_2b_ and C_4_ can be neglected.

The deviation between the measurement and the simulation results was determined using Equation (15). B corresponds to the calculated and M to the measured voltage Uc4Mean at the load. The squared difference of M and B was summed over an equal range of the root mean square voltage Uc2RMS from 0.7 V to 21 V with a step size of 10 mV and subsequently divided by the number of steps, N. For this calculation, M and B were piecewise linearly interpolated.

(15) $$S=\sqrt{\frac{1}{N}\sum^{\;}{\left(M-B\right)}^{2}}$$

## 3. Results and Discussion

First, we show the results for the circuit in Figure 1 with the circuit topology in Figure 2a having a linear capacitor C_2_ (47 nF, 200 V, C0G) and C_4_ (4.7 µF, 50 V). The capacitors C_2_ and C_4_ were defined as constant at 48 nF and 4.56 µF. The deviation S between the calculations with ANSYS/Mathcad and the measurements is shown in Table 1.

All selected calculation methods in Mathcad, regardless of the applied resolution, show a small deviation. A high consistency between calculations and measurements can also be achieved in ANSYS, except for the Euler method with constant step size and a resolution of 50 k and 500 k points and an adaptive step size, and the Adaptive Trapezoid-Euler method with constant step size and a resolution of 50 k points. Table 1 shows that in case of a linear capacitor C_2_ and C_4_, most calculation methods in ANSYS and all calculation methods in Mathcad lead to a high consistency between calculations and measurements. As an additional result, the memory consumption and computing time of the calculation methods used in Table 1 are shown in Table 2 and Table 3, respectively. The calculations were performed on a workstation HP Z250 (L8T12AV, Intel Xeon E3-1280 v5 (8M Cache, 3.70 GHz), 32 GB DDR4, 256 GB SSD, Windows 10 Pro 64-bit).

Table 2 shows that the calculations with Mathcad generally require less memory than with ANSYS, because the results obtained with Mathcad can be stored in binary format. For calculations with Mathcad and ANSYS with equal resolution of 50 k, 500 k and 5 M points and with constant step size, the memory consumption for calculations with ANSYS is about 5 times higher than with Mathcad. The memory consumption for the Euler and Adaptive Trapezoid-Euler methods (ANSYS) with an adaptive step size is about the same as for the calculation methods used in Mathcad at a resolution of 500 k points. On the other hand, the memory consumption for the Trapezoid method with an adaptive step size is about 1.5 times higher than with the calculations in Mathcad with a resolution of 5 M points. In terms of consistency and memory consumption, the Adams, Bulirsch–Stoer, Backward Differentiation Formula, Radau5 and the fourth-order Runge–Kutta method with constant and adaptive step size and a resolution of 50 k points (Mathcad) and the Trapezoid method with constant step size and a resolution of 50 k points (ANSYS) are most suitable.

Table 3 shows that the calculations with a resolution of 50 k points show the lowest computing time. It should also be noted that the Bulirsch–Stoer method with a resolution of 50 k, 500 k and 5 M points shows the highest computing time. In addition, the computing time with the Adams, Backward Differentiation Formula and Radau5 method changes only slightly at the different resolutions. In terms of consistency, memory consumption and computing time, the Adams, Radau5 and fourth-order Runge–Kutta method with a constant and adaptive step size and a resolution of 50 k points are most suitable in Mathcad and the Trapezoid method with a constant step size and a resolution of 50 k points is most suitable in ANSYS.

**Figure 5:**
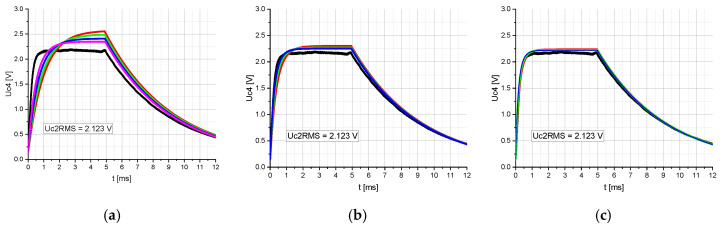
Representation of the measured (black) and calculated voltage Uc4 (Mathcad) over time t. Table 2. RMS was set to 2.123 V and the coupling factor k between the two inductances, L1 and L2, was set to: (**a**) 1% (red), 2% (green), 3% (blue), 4% (pink); (**b**) 5% (red), 6% (green), 7% (blue); (**c**) 8% (red), 9% (green), 10% (blue).

However, despite the small deviation, discrepancies in amplitude and time constants between the calculated and measured time-related voltage Uc4 were observed (Figure 5a). The same discrepancies have been observed in the previously published conference paper [19], although it was not clear whether they were due to the modeling of the two serially connected nonlinear capacitors, C_2a_ and C_2b_, or to another cause. Since these discrepancies occur in the case of both, a linear capacitor C_2_ and two serially connected nonlinear capacitors, C_2a_ and C_2b_, they cannot be assigned to the modeling of the nonlinear capacitors.

To find the root cause, the impact of the coupling factor k on the above-mentioned discrepancies was investigated. For this purpose, the calculations were performed with the circuit shown in Figure 1 having a linear capacitor C_2_ (Figure 2a) and C_4_. The coupling factor was varied from 1% to 10% in 1% steps and the amplitude of the sinusoidal voltage source was adjusted so that the voltage Uc2RMS was equal to 2.123 V. Figure 5 shows that an increasing coupling factor directly impacts the amplitude and time constant of the voltage Uc4. By changing the coupling factor between 1% and 4% (Figure 5a), the amplitude and time constant of voltage Uc4 change significantly. For coupling factors above 4%, the impact of the coupling factor on the amplitude and time constant becomes less significant (Figure 5b,c). At a coupling factor between 7% and 10%, the consistency between the calculated and measured time-related voltage curves Uc4 is highest (Figure 5c). Consequently, it should be ensured that the coupling factor is sufficiently high to achieve more accurate results even in the case of loose coupling.

**Figure 6:**
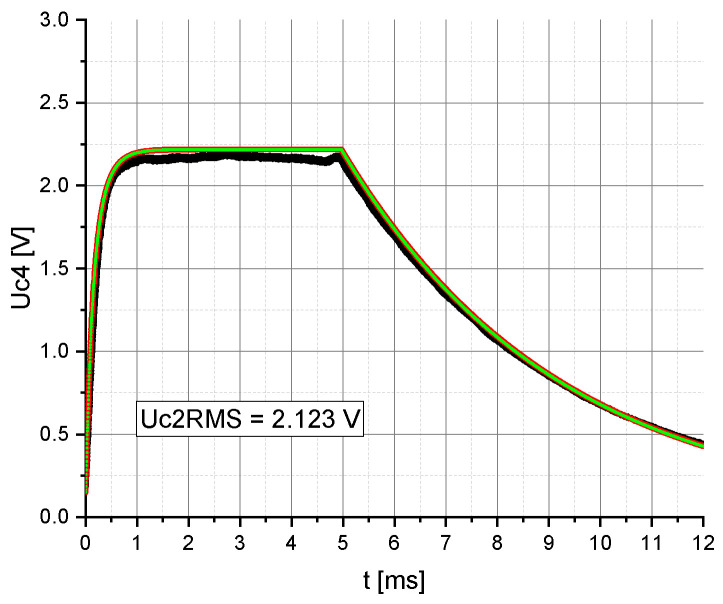
Representation of the measured (black) and calculated voltage (Mathcad) over time t. The voltage Uc2RMS was set to 2.123 V and the coupling factor, k, between the two inductances, L1 and L2, was set to 10%. Linear capacitor C4 (red), nonlinear capacitor C4 (green).

Finally, the impact of the nonlinear properties of the capacitor C_4_ on the model consistency was determined. The calculations were performed with the circuit in Figure 1 having a linear capacitor C_2_ (Figure 2a), a nonlinear capacitor C_4_ (Figure 4) and a coupling factor of 10%. According to Figure 6, the nonlinearity of the voltage rectifier capacitor C_4_ has no significant impact on the consistency of the model.

Based on these results, the calculations from Table 1 were repeated with a coupling factor k of 10%. Table 4 shows that increasing the coupling factor from 1% to 10% reduces the overall deviation, except for the Euler method with a resolution of 50 k points and a constant and adaptive step size. The reduction in deviation is especially noticeable in the Euler and Adaptive Trapezoid-Euler method. The deviation was reduced from 14.8 V to 0.3 V for the Euler method with constant step size and a resolution of 500 k points, and from 10.7 V to 0.3 V for the Adaptive Trapezoid-Euler method with constant step size and a resolution of 50 k points.

**Figure 7:**
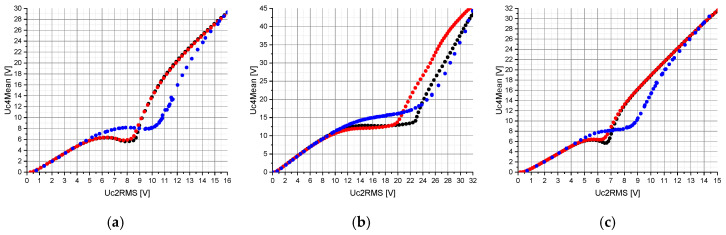
Representation of the measured (blue) and calculated voltage Uc4Mean with hysteresis losses (red) and without hysteresis losses (black) versus the voltage Uc2RMS. The calculations were performed for a circuit topology consisting of: (**a**) one nonlinear capacitor, C_2_ (see Figure 2a); (**b**) two serially connected nonlinear capacitors, C_2a_ and C_2b_, (see Figure 2b); (**c**) two nonlinear capacitors, C_2a_ and C_2b_, connected in parallel (see Figure 2c). Furthermore, the Adams method was used with a resolution of 50 k points and a coupling factor k of 10%.

The measurements and calculations in Figure 7 can be split into two parts. A part in which the relationship between Uc4Mean and Uc2RMS is linear and a part in which the nonlinear properties of the circuit topologies shown in Figure 2 are effective. Within the linear range, the consistency between calculations and measurements is high. However, Figure 7 shows that the threshold values to be reached by Uc2RMS for triggering the nonlinear behavior on Uc4Mean are lower in the calculations than in the measurements. The same observation was also made in the previous conference paper [19]. The impact of the hysteresis losses on the calculations is particularly noticeable in the circuit topology consisting of two serially connected nonlinear capacitors, C_2a_ and C_2b_ (Figure 7b). The value of Uc2RMS at which the voltage Uc4Mean increases changes from about 23 V to 20 V due to the hysteresis losses. The same behavior can also be observed with a nonlinear capacitor, C_2_, and two nonlinear capacitors, C_2a_ and C_2b_, connected in parallel (Figure 7a,c), in a range of Uc2RMS between about 6 V and 9 V. An interesting point in Figure 7 is that depending on the circuit topology used, an approximately constant range of Uc4Mean is achieved within a specific range of Uc2RMS. The range of Uc2RMS in which Uc4Mean is approximately constant and the slope of Uc4Mean within this range are defined by the circuit topology of nonlinear capacitors.

Despite the increase in the coupling factor k from 1% to 10% and the implementation of hysteresis losses, the calculations deviate from the measurements for higher values of Uc2RMS. A possible explanation would be that the measurement of the nonlinear capacitors used in this work, according to Section 2.1, is no longer valid for higher AC voltages [25,26,27].

## 4. Conclusions

This paper describes the optimization steps for modeling the nonlinear properties of ferroelectric materials in ceramic capacitors in terms of consistency, memory consumption and computing time. It turned out that the coupling factor k directly impacts the consistency between simulation and measurement. Particular attention should be paid to ensure a sufficiently high coupling factor k even in the case of loose coupling. A coupling factor between 7% and 10% should be adequate to properly model the time constant of the inductive power transmission system.

In addition, it was found that the consideration of the nonlinear properties of the capacitor C_4_ does not significantly improve the model, but increases the computing time. Therefore, with regard to consistency and computing time, we recommend neglecting the nonlinear properties of the capacitor C_4_ for further modeling purposes.

Based on the results of the previously published conference paper, it was concluded that the Trapezoid method with a constant step size and a resolution of 500 k points and with an adaptive step size is most suitable in ANSYS [19]. Considering the computing time and memory consumption in Table 2 and Table 3, the Trapezoid method with a constant step size and a resolution of 500 k points should be preferred.

A high consistency between calculations and measurements was achieved in Mathcad using the Adams, Bulirsch–Stoer, Backward Differentiation Formula, Radau5, and fourth-order Runge–Kutta method with an adaptive step size and a resolution of 50 k points. The calculations in Mathcad with a resolution of 50 k points show the lowest memory consumption (Table 2). With regard to the computing time, we recommend using the Adams method in the first place and the Backward Differentiation Formula and Radau5 method as an alternative (Table 3).

Based on these results, a simulation model for modeling ferroelectric materials in ceramic capacitors is now available that exhibits high consistency and efficiency in terms of computing time and memory consumption. Nevertheless, the simulation model is limited to lower AC voltages across the circuit topology of nonlinear capacitors. In order to expand the application of the model to higher AC voltages, it is necessary in the next step to characterize the voltage dependence of ceramic capacitors for large signals. Furthermore, the impact of the manufacturing tolerances of ferroelectric capacitors on the robustness of the collective nonlinear dynamics of the proposed meaningful circuit topology should be investigated [28].

We plan to use this simulation model in combination with various optimization algorithms to establish a frugal circuit topology with nonlinear components for the realization of a closed-loop current control. This will increase the degree of miniaturization in electronic implants because there will be no need to use dedicated sensors or other active electronic components. Electronic implants with inductive power supply, such as retinal implants [1,6,7,9], cochlear implants [29,30], and the hypoglossal nerve stimulator GenioTM (Nyxoah SA, Mont-Saint-Guibert, Belgium) [31] would be particularly suitable for this purpose.

## Figures and Tables

**Table 1:** Deviations between the measured and calculated voltage Uc4Mean in a range of Uc2RMS from 0.7 V to 21 V at a coupling factor k of 1%.

Method	50 k Points	500 k Points	5 M Points
Adams	0.5 V	0.5 V	0.5 V
Bulirsch–Stoer	0.3 V	0.3 V	0.3 V
Runge–Kutta ^1^	0.5 V	0.5 V	0.5 V
Runge–Kutta ^2^	0.3 V	0.3 V	0.3 V
BDF ^4^	0.5 V	0.5 V	0.5 V
Radau5	0.5 V	0.5 V	0.5 V
Euler ^1^	15.5 V	14.8 V	0.5 V
Trapezoid ^1^	0.5 V	0.6 V	0.6 V
ATE ^1,3^	10.7 V	0.6 V	0.6 V
Euler ^2^	15.5 V		
Trapezoid ^2^	0.6 V		
ATE ^2,3^	0.5 V		

^1^ With constant step size; ^2^ With variable step size; ^3^ Adaptive Trapezoid-Euler; ^4^ Backward Differentiation Formula.

**Table 2:** Memory consumption of the selected calculation methods in Mathcad and ANSYS summed up over 100 independent runs.

Method	50 k Points	500 k Points	5 M Points
Adams	0.23 GB	2.23 GB	22.3 GB
Bulirsch–Stoer	0.23 GB	2.23 GB	22.3 GB
Runge–Kutta ^1^	0.23 GB	2.23 GB	22.3 GB
Runge–Kutta ^2^	0.23 GB	2.23 GB	22.3 GB
BDF ^4^	0.23 GB	2.23 GB	22.3 GB
Radau5	0.23 GB	2.23 GB	22.3 GB
Euler ^1^	1.18 GB	11.7 GB	118 GB
Trapezoid ^1^	1.18 GB	11.7 GB	117 GB
ATE ^1,3^	1.18 GB	11.7 GB	117 GB
Euler ^2^	2.84 GB		
Trapezoid ^2^	34.9 GB		
ATE ^2,3^	2.33 GB		

^1^ With constant step size; ^2^ With variable step size; ^3^ Adaptive Trapezoid-Euler; ^4^ Backward-Differentiation-Formula.

**Table 3:** Computing time (hh:mm:ss) of the selected calculation methods in Mathcad summed up over 80 independent runs.

Method	50 k Points	500 k Points	5 M Points
Adams	00:03:17	00:03:49	00:10:03
Bulirsch–Stoer	00:16:17	01:12:57	08:56:55
Runge–Kutta ^1^	00:01:48	00:15:41	02:42:50
Runge–Kutta ^2^	00:07:58	00:42:35	06:39:06
BDF ^4^	00:12:33	00:12:48	00:19:13
Radau5	00:07:42	00:08:33	00:13:11
Euler ^1^	00:01:51	00:06:29	00:44:27
Trapezoid ^1^	00:01:45	00:06:17	00:52:25
ATE ^1,3^	00:04:43	00:14:30	00:43:16
Euler ^2^	00:02:49		
Trapezoid ^2^	00:21:13		
ATE ^2,3^	00:02:04		

^1^ With constant step size; ^2^ With variable step size; ^3^ Adaptive Trapezoid-Euler; ^4^ Backward-Differentiation-Formula.

**Table 4:** Deviations between the measured and calculated voltage Uc4Mean in a range of Uc2RMS from 0.7 V to 21 V at a coupling factor k of 10%.

Method	50 k Points	500 k Points	5 M Points
Adams	0.3 V	0.3 V	0.3 V
Bulirsch–Stoer	0.3 V	0.3 V	0.3 V
Runge–Kutta ^1^	0.4 V	0.3 V	0.3 V
Runge–Kutta ^2^	0.3 V	0.3 V	0.3 V
BDF ^4^	0.3 V	0.3 V	0.3 V
Radau5	0.3 V	0.3 V	0.3 V
Euler ^1^	15.5 V	0.3 V	0.4 V
Trapezoid ^1^	0.4 V	0.4 V	0.4 V
ATE ^1,3^	0.3 V	0.4 V	0.4 V
Euler ^2^	15.1 V		
Trapezoid ^2^	0.4 V		
ATE ^2,3^	0.4 V		

^1^ With constant step size; ^2^ With variable step size; ^3^ Adaptive Trapezoid-Euler; ^4^ Backward Differentiation Formula.

## References

[1] Bloch E., Luo Y., da Cruz L. (2019). Advances in retinal prosthesis systems. Ther. Adv. Ophthalmol..

[2] Stingl K., Bartz-Schmidt K.U., Besch D., Chee C.K., Cottriall C.L., Gekeler F., Groppe M., Jackson T.L., MacLaren R.E., Koitschev A. (2015). Subretinal Visual Implant Alpha IMS—Clinical trial interim report. Vision Res..

[3] Mertens A., Raedt R., Gadeyne S., Carrette E., Boon P., Vonck K. (2018). Recent advances in devices for vagus nerve stimulation. Expert Rev. Med. Devices.

[4] Wray C.M., Thaler E.R. (2016). Hypoglossal nerve stimulation for obstructive sleep apnea: A review of the literature. World J. Otorhinolaryngol. Head Neck Surg..

[5] Wolter T., Kieselbach K. (2012). Cervical spinal cord stimulation: An analysis of 23 patients with long-term follow-up. Pain Physician.

[6] Finn A.P., Grewal D.S., Vajzovic L. (2018). Argus II retinal prosthesis system: A review of patient selection criteria, surgical considerations, and post-operative outcomes. Clin. Ophthalmol..

[7] Gekeler F., Szurman P., Grisanti S., Weiler U., Claus R., Greiner T.-O., Völker M., Kohler K., Zrenner E., Bartz-Schmidt K.U. (2007). Compound subretinal prostheses with extra-ocular parts designed for human trials: Successful long-term implantation in pigs. Graefes Arch. Clin. Exp. Ophthalmol..

[8] Laube T., Brockmann C., Roessler G., Walter P., Krueger C., Goertz M., Klauke S., Bornfeld N. (2012). Development of surgical techniques for implantation of a wireless intraocular epiretinal retina implant in Göttingen minipigs. Graefes Arch. Clin. Exp. Ophthalmol..

[9] Roessler G., Laube T., Brockmann C., Kirschkamp T., Mazinani B., Goertz M., Koch C., Krisch I., Sellhaus B., Trieu H.K. (2009). Implantation and explantation of a wireless epiretinal retina implant device: Observations during the EPIRET3 prospective clinical trial. Invest. Ophthalmol. Vis. Sci..

[10] Doshi P.K. (2011). Long-term surgical and hardware-related complications of deep brain stimulation. Stereotact. Funct. Neurosurg..

[11] Müller C., Koch T. (2018). Control Circuit for a Base Station for Transmitting Energy to a Receiver by Means of an Electric Resonant Circuit, Evaluation Device, Method and Computer Program. U.S. Patent.

[12] Seo D., Carmena J.M., Rabaey J.M., Alon E., Maharbiz M.M. (2013). Neural Dust: An Ultrasonic, Low Power Solution for Chronic Brain-Machine Interfaces. arXiv.

[13] Kolberg I., Shmilovitz D., Ben-Yaakov S.S. (2018). Ceramic capacitor controlled resonant LLC converters. Proceedings of the 2018 IEEE Applied Power Electronics Conference and Exposition (APEC).

[14] Borafker S., Drujin M., Ben-Yaakov S.S. (2018). Voltage-Dependent-Capacitor Control of Wireless Power Transfer (WPT). Proceedings of the 2018 IEEE International Conference on the Science of Electrical Engineering in Israel (ICSEE).

[15] Ben-Yaakov S., Zeltser I. (2018). Ceramic capacitors: Turning a deficiency into an advantage. Proceedings of the 2018 IEEE Applied Power Electronics Conference and Exposition (APEC).

[16] Tishechkin S., Ben-Yaakov S. (2019). Adaptive Capacitance Impedance Matching (ACIM) of WPT Systems by Voltage Controlled Capacitors. Proceedings of the 2019 IEEE PELS Workshop on Emerging Technologies: Wireless Power Transfer (WoW).

[17] Zeng H., Peng F.Z. (2018). Non-linear capacitor based variable capacitor for self-tuning resonant converter in wireless power transfer. Proceedings of the 2018 IEEE Applied Power Electronics Conference and Exposition (APEC).

[18] Zhang L., Ngo K. (2018). A Voltage-Controlled Capacitor with Wide Capacitance Range. Proceedings of the 2018 IEEE Energy Conversion Congress and Exposition (ECCE).

[19] Olsommer Y., Ihmig F.R., Müller C. (2020). Modeling the Nonlinear Properties of Ferroelectric Materials in Ceramic Capacitors for the Implementation of Sensor Functionalities in Implantable Electronics. Proceedings.

[20] Brindley G.S. (1970). Sensations produced by electrical stimulation of the occipital poles of the cerebral hemispheres, and their use in constructing visual prostheses. Ann. R. Coll. Surg. Engl..

[21] Karny H. (1975). Clinical and physiological aspects of the cortical visual prosthesis. Surv. Ophthalmol..

[22] Lewis P.M., Rosenfeld J.V. (2016). Electrical stimulation of the brain and the development of cortical visual prostheses: An historical perspective. Brain Res..

[23] Mashiach A., Mueller C. (2016). Head Pain Management Device Having an Antenna. U.S. Patent.

[24] Jadli U., Mohd-Yasin F., Moghadam H.A., Nicholls J.R., Pande P., Dimitrijev S. (2020). The Correct Equation for the Current through Voltage-Dependent Capacitors. IEEE Access.

[25] Institute of Electrical and Electronics Engineers, International Symposium on Electromagnetic Compatibility, EMC Europe (2013). International Symposium on Electromagnetic Compatibility (EMC Europe), 2–6 September 2013, Brugge, Belgium.

[26] Cokkinides G.J., Becker B. (2000). Modeling the effects of nonlinear materials in ceramic chip capacitors for use in digital and analog applications. IEEE Trans. Adv. Packag..

[27] Zhang L., Ritter A., Nies C., Dwari S., Guo B., Priya S., Burgos R., Ngo K. (2017). Voltage-Controlled Capacitor—Feasibility Demonstration in DC–DC Converters. IEEE Trans. Power Electron..

[28] Chikhaoui K., Bitar D., Kacem N., Bouhaddi N. (2017). Robustness Analysis of the Collective Nonlinear Dynamics of a Periodic Coupled Pendulums Chain. Appl. Sci..

[29] Hochmair I., Nopp P., Jolly C., Schmidt M., Schößer H., Garnham C., Anderson I. (2006). MED-EL Cochlear Implants: State of the Art and a Glimpse Into the Future. Trends Amplif..

[30] Lenarz T. (2018). Cochlear implant—state of the art. GMS Curr. Top. Otorhinolaryngol. Head Neck Surg..

[31] Eastwood P.R., Barnes M., MacKay S.G., Wheatley J.R., Hillman D.R., Nguyên X.-L., Lewis R., Campbell M.C., Pételle B., Walsh J.H. (2020). Bilateral hypoglossal nerve stimulation for treatment of adult obstructive sleep apnoea. Eur. Respir. J..

